# Editorial: Uncommon or rare forms of diabetes: from diagnosis to management

**DOI:** 10.3389/fmed.2026.1831564

**Published:** 2026-04-10

**Authors:** Valeria Grancini, Andrea Mario Bolla

**Affiliations:** 1 Endocrinology Unit, Fondazione Istituto di Ricovero e Cura a Carattere Scientifico Ca' Granda Ospedale Maggiore Policlinico di Milano, Milano, Italy; 2 Diabetology Unit, Guglielmo da Saliceto Hospital, Piacenza, Italy

**Keywords:** cystic fibrosis related diabetes, genetic diabetes, immune check point inhibitor, monogenic diabetes (MODY), rare diabetes

Diabetes mellitus encompasses a heterogeneous spectrum of disorders, ranging from classical type 1 (T1D) and type 2 diabetes (T2D) to rare or atypical forms characterized by unique genetic, autoimmune, iatrogenic, or syndromic etiologies. While T1D and T2D together account for the vast majority of diabetes cases, a significant minority arise from uncommon mechanisms that demand tailored diagnostic and therapeutic approaches [Ozsu; (1, 2)].

Rare diabetes types, including monogenic syndromes, mitochondrial and syndromic forms, and therapy-induced phenotypes, collectively represent a clinically relevant subset of patients who may easily be misdiagnosed or suboptimally treated without precise characterization (3, 4).

This Research Topic brings together 10 contributions that collectively illustrate the clinical, molecular, and therapeutic complexity of these rare diabetes phenotypes and emphasize the growing importance of precision medicine in diabetes care.

Rare syndromes highlight the intricate interplay between genetics and metabolism. In the pediatric context, Pietrzykowska et al. conducted a systematic review on cystic fibrosis–related diabetes (CFRD) in patients receiving CFTR modulators, reporting that these therapies may improve glucose tolerance and insulin secretion in children and adolescents with preserved β-cell function, although evidence remains limited and variable. This signals the need for larger pediatric trials with standardized metabolic outcomes to determine the long-term glycemic benefits of CFTR modulation (Pietrzykowska et al.).

At a molecular level, monogenic diabetes provides mechanistic insight that informs both diagnosis and therapy. Rare monogenic variants account for approximately 1–5% of all diabetes cases, and accurate recognition is crucial for implementing effective personalized management [Ozsu; (2)]. In this context, mitochondrial dysfunction and endoplasmic reticulum (ER) stress have emerged as key convergent pathways impairing β-cell function, disrupting insulin secretion, and contributing to glycemic dysregulation [Caretto et al.; Ruiz-Urbaez et al.; Gulisano et al.; (5–12)]. Ozsu summarize current understanding of these mechanisms, emphasizing how mitochondrial defects and ER stress can serve as diagnostic clues and targets for future therapeutic strategies.

Maturity-Onset Diabetes of the Young (MODY) exemplifies the clinical and genetic heterogeneity of monogenic diabetes. Misdiagnosis as T1D, T2D, or gestational diabetes remains common, often delaying optimal therapy by years (Ruiz-Urbaez et al.; Gulisano et al.). In the Research Topic, the study from a Latin American tertiary center highlights that whole-exome sequencing is crucial to identify pathogenic variants and reclassify diabetes type accurately, enabling individualized therapy (Ruiz-Urbaez et al.). Complementing this, Gulisano et al. describe an adolescent with an HNF4A variant whose diagnosis of MODY1 allowed a successful transition from insulin to sulfonylurea therapy, greatly improving glycemic control and quality of life.

Clinical vigilance remains essential, as atypical presentations may serve as early clues. Syndromic features may precede or signify complex disease. For example, a case of Klinefelter syndrome presenting with diabetic ketoacidosis underscores how chromosomal abnormalities may underlie metabolic disturbances and should prompt comprehensive diagnostic evaluation (4). Dermatologic manifestations of diabetes are also frequent clinical indicators: up to one-third of patients can develop skin lesions such as necrobiosis lipoidica, bullosis diabeticorum, and diabetic dermopathy, which can precede systemic complications and signal underlying metabolic dysregulation [Pietrzykowska et al.; (5)]. Early recognition facilitates timely diagnostic testing and targeted therapy, enhancing outcomes and quality of life [Pietrzykowska et al.; (5)].

Pregnancy adds further complexity to diagnosis and management. While hyperglycemia during gestation is most commonly attributable to gestational diabetes, a portion of cases reflects underlying autoimmune or monogenic forms. Autoantibody screening can identify women at risk for progression to T1D after pregnancy, guiding individualized insulin therapy and follow-up [Caretto et al.; (3)]. In this vein, two contributions in this Topic address type 1 diabetes diagnosed during pregnancy and diabetic mellitus associated with Wolfram Syndrome type 1 (WS1). The latter highlights how reproductive outcomes in WS1, including successful cases managed with hybrid closed-loop insulin pumps, require coordinated care integrating advanced technologies with genetic insight (Caretto et al.).

Therapeutic innovation continues to expand the diabetes care landscape. Continuous intraperitoneal insulin infusion (CIPII) has emerged as a potent approach in patients with unstable T1D and subcutaneous insulin resistance, promoting more physiologic hepatic insulinization and reducing post-prandial glycemic variability compared with traditional subcutaneous insulin therapy (2). Concurrently, hybrid closed-loop systems are increasingly applied to complex patients, bridging the gap between physiological insulin requirements and real-world glycemic challenges (Caretto et al.).

Immune checkpoint inhibitor–related diabetes mellitus (ICI-DM) represents an important example of iatrogenic diabetes arising from cancer immunotherapy. Characterized by fulminant hyperglycemia, frequent ketoacidosis, and autoantibody positivity, ICI-DM underscores how genetic predispositions and immune modulation intersect to trigger rapid β-cell failure (5). Early recognition of ICI-DM and timely insulin initiation are critical to prevent acute metabolic decompensation, while ongoing research seeks to clarify the optimal approach to cancer immunotherapy continuation in affected patients (5).

Across these diverse presentations, a consistent theme emerges: early and precise diagnosis informed by genetics, careful clinical evaluation, and molecular insights is indispensable. Precision medicine approaches—leveraging modern sequencing technologies, advanced insulin delivery systems, and tailored pharmacologic interventions—offer the potential to optimize clinical outcomes and enhance quality of life for patients with rare diabetes forms [Ozsu; Pietrzykowska et al.; Caretto et al.; Ruiz-Urbaez et al.; Gulisano et al.; (1, 2, 4–6)]. As diabetes prevalence continues to rise globally (8), the ability to recognize subtle clinical clues and apply individualized therapeutic strategies will be central to effective care (1, 4, 13).

The studies collected in this Research Topic underscore the diversity and complexity of rare diabetes forms. Clinicians must look beyond the traditional dichotomy of “type 1 or type 2” and maintain a high index of suspicion for alternative diagnoses (7); this will enable early genetic and molecular characterization, integration of novel interventions, and application of advanced technologies (9, 11). Moving forward, larger multicenter trials and collaborative registries are essential to refine diagnostic algorithms, elucidate epidemiological patterns, and assess the long-term efficacy of targeted therapies [Ozsu; Ruiz-Urbaez et al.; (1, 4, 10)]. Ultimately, these advances pave the way for a precision medicine framework in diabetes care, ensuring that even the rarest forms receive optimal, individualized management [Ozsu; Pietrzykowska et al.; Caretto et al.; Ruiz-Urbaez et al.; Gulisano et al.; (1, 4, 5)].
